# Parents’ Perspectives and Processes During Weaning for Preterm‐Born Children: A Qualitative Study in Japan

**DOI:** 10.1155/jonm/5519814

**Published:** 2026-08-17

**Authors:** Maki Fujitsuka, Shingo Ueki, Seiichi Morokuma

**Affiliations:** ^1^ Department of Health Sciences, Graduate School of Medical Sciences, Kyushu University, Fukuoka, Japan, kyushu-u.ac.jp

**Keywords:** experience, low birth weight infant, parent, preterm infant, weaning

## Abstract

**Background:**

Feeding difficulties are common among preterm‐born children. During the weaning process, incorporating parents’ perspectives into support systems is essential to enhance family‐centered care.

**Aim:**

This study aimed to clarify parents’ experiences during the weaning period of preterm‐born children by exploring how they perceive their children’s condition and navigate the process.

**Methods:**

Nine parents who were weaning their preterm‐born children under 24 months of corrected age were included. Semi‐structured interviews were conducted in Japan between August and October 2024. The qualitative data were then analyzed using content analysis.

**Results:**

All participants were mothers. Their children’s mean gestational age at birth was 29.7 ± 4.4 weeks, and the mean corrected age at the interview was 15 ± 4 months. Across challenges, successes, and support stages, seven categories were identified: challenges—(1) Difficulties in deviating from standard weaning practices, (2) Impatience with children’s growth and development; successes—(3) Weaning based on children’s actual feeding behavior, (4) Cooperation with family and experienced caregivers; support—(5) Need for timely access to professional consultation, (6) Exploration of approaches tailored to children’s condition with professionals, and (7) Relief and confidence through collaboration with professionals. Parents were unable to progress age‐based weaning milestones and required support from healthcare professionals. Parents focused on their children’s actual feeding behaviors to navigate at their child’s own pace.

**Conclusion:**

Challenges specific to prematurity during weaning led parents to explore approaches tailored to each child’s needs. Although they experienced impatience regarding their children’s growth, support from healthcare professionals provided reassurance.

**Implications for Nursing Management:**

To provide family‐centered care during the weaning process, nursing management should establish a coordinated support system that spans the neonatal intensive care unit (NICU) and community healthcare settings. This system should include discharge planning, follow‐up assessments of feeding development, and nurse education on the long‐term developmental needs of preterm‐born children. In addition, accessible consultation pathways should be established to enable parents to seek timely advice and facilitate interdisciplinary collaboration tailored to the needs of each child and family.

## 1. Introduction

In 2023, Japan recorded approximately 42,000 preterm infants (born before 37 weeks of gestation), representing approximately 5.7% of all live births [1]. This rate has remained relatively stable in recent years, consistent with those observed in many other countries. The earlier a preterm infant is born, the fewer opportunities they get to develop their sucking and swallowing abilities in utero, resulting in immature oral motor function [2, 3]. Given that feeding is a complex behavior regulated by the central nervous system and shaped by developmental processes [4], prematurity and medical interventions in NICU can interfere with infants’ feeding skill development [5]. Furthermore, medical complications such as chronic lung disease, necrotizing enterocolitis, and hypotonia, can delay and disrupt feeding development [6, 7].

Insufficient development of oral motor function during the breastfeeding period may impact later feeding, including food intake, chewing, and swallowing, which require more complex oral motor coordination. Preterm‐born children exhibit reduced abilities in feeding skills such as chewing and swallowing compared with full‐term children [8]. Between 6 and 12 months old, 43% of preterm‐born children display feeding difficulties, including coughing and gagging; this percentage is 3.2 times higher than that of full‐term children [9]. Even after 12 months old, 25% continue to experience such difficulties, representing 2.66 times the prevalence observed in full‐term children.

Weaning is also referred to as complementary feeding, which is the process of providing foods in addition to milk when breast milk or milk formula alone are no longer adequate to meet nutritional requirements [10]. Given that feeding develops within a critical period up to 2 years of age [11], the early introduction of oral feeding in NICU is considered an essential intervention to prepare preterm‐born children for transitioning to full oral feeding [12]. In this context, providing early support in the weaning process is crucial for parents who care for preterm‐born children at home.

Parents of preterm‐born children often struggle with the weaning process, because it frequently does not progress smoothly. Compared with parents of full‐term children, they report higher levels of anxiety and stress related to weaning [13, 14]. Furthermore, unlike parents of full‐term children, they do not exhibit the same developmental progression in behaviors that promote feeding interactions during the weaning process [15]. When weaning becomes particularly difficult for preterm‐born children, parents adopt a more forceful approach, disregarding signals and interrupting ongoing behavior [16]. Hence, parents of preterm‐born children require support tailored to their specific challenges during the weaning process. However, it remains unclear how parents navigate this process in the face of such difficulties.

Studies on weaning in preterm‐born children mainly focus on the effects of the timing of weaning initiation on physical growth [17–19]. Corrected age, which is calculated according to the expected date of delivery rather than the actual date of birth, is commonly used. However, existing guidelines currently lack detailed instructions on intervention strategies or structured support programs for preterm‐born children [19]. Developing appropriate support systems that can be implemented in practical settings, such as NICU follow‐up clinics and community health centers, is crucial.

Family‐centered care is a key principle in pediatric healthcare; thus, the American Academy of Pediatrics [20] emphasizes the need to facilitate research on the development of interventions and systems to support it. In neonatal care, previous qualitative studies have explored parents’ experiences to inform family‐centered practice, healthcare system design, and the education of healthcare professionals [21–23]. To enhance the quality of family‐centered care during the weaning process, healthcare providers need to first understand parents’ perspectives and experiences, identify the challenges and successes they encounter, and use these insights to inform the development of structured support systems. This study aimed to clarify parents’ experiences during the weaning period of their preterm‐born children by exploring how they perceive their children’s condition and navigate the process. The findings provide foundational qualitative insights to support the development and evaluation of future intervention programs and systems.

## 2. Methods

### 2.1. Study Design

This study used a qualitative descriptive design to describe parents’ experiences in caring for their preterm‐born children in Japan. The Consolidated Criteria for Reporting Qualitative Research (COREQ) guided the reporting for this study [24].

### 2.2. Participants

Parents of preterm‐born children were recruited using purposive sampling. The eligibility criteria for parent participants were as follows: (1) had a preterm‐born child under 24 months of corrected age, (2) had been weaning their child for more than 2 months, and (3) conducted weaning primarily within the family. Conversely, parents with foreign nationality who practice food cultures, including weaning, that are different from those in Japan were excluded. Grandparents were not included, as they are usually not considered primary caregivers in Japan, where nuclear families are increasingly common [25].

### 2.3. Data Collection

In this community‐based study, parents who belonged to the family support groups for parents of preterm‐born children in Japan were enrolled between August and October 2024. These groups were formed across various regions in Japan, including both urban and suburban areas. The first author asked the support group heads to introduce parents who met the eligibility criteria. With the parents’ permission, the heads shared their email addresses, and the first author contacted them directly via email. This first author was involved as a volunteer nurse in one of the support groups and had previous contact with some of the parents belonging to the group. However, none of the parents who participated in this study were personally acquainted with the author.

Data were collected using semi‐structured interviews conducted by the first author. After a relevant literature review on weaning preterm‐born children, the interview guide was developed as follows: (1) How did you start weaning? (2) What was difficult about weaning? (3) What did you think went well with weaning? (4) What strategies did you use to proceed with weaning? (5) How did you assess the weaning process? and (6) What support did you receive regarding weaning? Participant characteristics, such as parent’s age, child’s age, and complications, were collected. Socioeconomic factors, such as parent’s income and locality, were not collected in this study.

Interviews were conducted either in a private room or via an online meeting, and participants’ children were present during some sessions. All interviews were recorded using a digital voice recorder, and field notes were also taken. At the beginning of each interview, the first author engaged in light conversation and indicated having personal experience with weaning as a parent to foster a sense of familiarity and encourage open communication.

### 2.4. Data Analysis

Audio recordings of the interviews were transcribed verbatim using Microsoft Excel 365. To systematically and objectively describe the phenomenon, an inductive content analysis was conducted [26]. The transcripts were read repeatedly and segmented into 619 meaning units through coding. Similar codes were grouped into subcategories, which were then abstracted into categories [27]. The first author conducted this analytical process without using any qualitative analysis software.

### 2.5. Trustworthiness

The trustworthiness of this qualitative content analysis was ensured by addressing the concepts of credibility, dependability, transferability, and confirmability [26]. The following strategies were employed to ensure methodological rigor. For credibility, purposive sampling was employed according to the study’s purpose and eligibility criteria, which were first explained to the heads of family support groups, who then facilitated the recruitment of targeted participants. The sample size was determined according to the studied phenomena’s complexity and data quality [27]. Given the use of purposive sampling and content analysis, code saturation was expected to be achieved with approximately nine interviews [28]. In the present study, data saturation was reached after interviews with nine participants, as no new information emerged. During the analysis, the categories were discussed and revised until consensus was reached among five qualitative researchers with expertise in pediatric care.

To ensure dependability, a researcher experienced in neonatal nursing conducted interviews using a semi‐structured guide. An audit trail documenting the data collection and analysis processes was maintained and reviewed within the research team. Regarding transferability, we recruited participants from multiple family support groups located in different regions to include those participants with diverse caregiving experiences and sociocultural backgrounds. We have provided detailed descriptions of the data collection procedures, participant characteristics, and research environment so that readers can evaluate the relevance of the findings to other contexts. As for confirmability, illustrative quotations from the transcribed data have been presented, thereby substantiating the analytical process and demonstrating clear links between participants’ narratives and the subcategories derived from the data.

### 2.6. Ethical Considerations

This study was approved by the Ethical Review Committee of Kyushu University Hospital (Approval No.: 24107). The first author provided both oral and written explanations to all participants, outlining the study’s purpose, procedures, voluntary participation, the right to withdraw at any time, and anonymity and confidentiality assurances. Interviews were conducted only after obtaining participants’ written informed consent. All collected data were anonymized using participant identification codes and securely stored with password protection. As a token of appreciation, participants received a 3000‐yen shopping voucher after the interview.

## 3. Results

Of the 10 parents who were approached through family support groups, nine parents agreed to participate in the study and completed the interview (response rate: 90%). Their background data are shown in Table 1. All participants were mothers, with a mean age of 37.3 ± 4.2 years. Their children’s mean gestational age at birth was 29.7 ± 4.4 weeks, and their mean corrected age at the interview was 15 ± 4 months. The average interview duration was 50 min (range: 41–66 min).

**TABLE 1:** Participant characteristics.

	*n* (%)/mean ± SD (range)
Parent	
Mother	9 (100%)
Age (years)	37.3 ± 4.2 (32–44)
Child	
Gestational age at birth (weeks)	29.7 ± 4.4 (23.4–35.9)
< 28 weeks	4 (44%)
29–32 weeks	2 (22%)
33–36 weeks	3 (33%)
Birth weight (gram)	1136 ± 600
First child	7 (78%)
Corrected age at interview (months)	15 ± 4 (9–22)
Dysphagia/Tube feeding at interview	0
Home oxygen therapy at interview	0
Daycare center	3 (33%)

From the analysis of interviews on parental experiences during the weaning of preterm‐born children, three stages were identified: challenges (Table 2), successes (Table 3), and support (Table 4). Across these stages, seven categories were identified. The challenges stage included two categories: (1) Difficulties in deviating from standard weaning practices, (2) Impatience with children’s growth and development. The successes stage included two categories: (3) Weaning based on children’s actual feeding behavior, (4) Cooperation with family and experienced caregivers. The support stage included three categories: (5) Need for timely access to professional consultation, (6) Exploration of approaches tailored to children’s condition with professionals, and (7) Relief and confidence through collaboration with professionals.

**TABLE 2:** Challenges: categories and subcategories.

Categories/subcategories	Representative codes
*Difficulties in deviating from standard weaning practices/*	
Difficulty deciding to start weaning based on criteria such as age or physical development	*“Since he was a little delayed in sitting up, he could sit in a chair with support, but he still couldn’t sit up on his own at 7 months of corrected age.” (Participant G)*
Difficulty progressing to chewing or swallowing solid foods	*“Because the food had some texture, I think he couldn’t mash it well while swallowing. During a single meal, he would gag four or five times, which became more frequent.” (Participant I)*
Anxiety during children’s vomiting or refusal during feeding	*“I was really struggling because he wouldn’t eat; he wouldn’t open his mouth or would just push the food out.” (Participant D)*
Inability to progress according to corrected age	*“Now at 9 months of corrected age, he is about 2 months behind in development. I struggled a lot, whether I should follow the typical 9-month feeding stage or adjust the weaning process to match their developmental age.” (Participant A)*
Concern about effects of ingredients or amounts unsuitable for corrected age on children’s digestion or allergies	*“When I looked at the reference books, there were clear stages showing what babies should be eating at each month of age. But since I started weaning 2 months later than that, everything felt out of sync. I kept wondering whether he was really getting the nutrients he needed for current age.” (Participant H)*
Difficulty judging whether information from peer support or internet is appropriate for children	*“Some experienced parents at the family support group told me that some babies didn’t like pureed textures and that my baby would start eating once he could do finger-feeding. So, I tried different things, like making the food a bit thicker or firmer. But in my baby’s case, none of it worked at all.” (Participant F)*

*Impatience with children’s growth and development/*	
Anxiety about whether children are gaining weight steadily along growth chart	*“I was hoping he would at least reach the middle of the growth chart; that would have made me feel most reassured.” (Participant C)*
Depression arising from comparisons with full‐term children’s development	*“The baby food class was held in a group setting, and once I went, I found myself comparing him with other children around the same age. That made me decide to stop going.” (Participant G)*
Burden of feeding to support children’s growth	*“I really wanted him to grow, so I kept thinking, ‘If he won’t eat or drink, what else can I do?’” (Participant F)*

**TABLE 3:** Successes: categories and subcategories.

Categories/subcategories	Representative codes
*Weaning based on children’s actual feeding behavior/*	
Waiting for moment to start, trying not to miss children’s motivation to eat	*“When he started showing interest in what we were eating by asking things like, ‘What’s that?’, I decided to start from there.” (Participant E)*
Relief that children do not dislike eating itself	*“When I started, things went really well for about the first month. He finished everything at each meal, and I felt like, ‘I’m really glad I started.’” (Participant F)*
Identifying indicators other than age and valuing children’s pace	*“I realized I didn’t have to force myself just because he was a certain number of months old. It was okay to keep the food in smaller pieces or softer textures based on his needs.” (Participant I)*
Adjusting food texture and taste to what children can eat rather than age	*“He had completely stopped eating the plain, natural-tasting foods typical of the early weaning stage, so I kept the stronger flavors from the mid-stage but went back to the smoother textures of the early stage.” (Participant B)*
Prioritizing children’s eating behavior over weight, with sufficient milk intake when weight gain is adequate	*“I was told that his weight was progressing well for his corrected age, so more than the numbers, I felt that the most important thing was how he was actually eating.” (Participant A)*

*Cooperation with family and experienced caregivers/*	
Uncertainty about how to start, leading to consultation with guidelines and experienced caregivers	*“I wasn’t sure if I could start weaning the same way as with typical children, but I decided to think it through myself and follow a baby food book as a reference.” (Participant I)*
Proceeding in consultation with family	*“I also consulted with my parent who lives nearby, and they told me, ‘It’s okay to go at his own pace.’” (Participant G)*
Sharing feeding difficulties in peer support settings, leading to positive reframing	*“When I consulted the family support group saying, ‘I’m struggling because he hardly eats,’ everyone shared photos of times when their children also didn’t eat much, and I was able to hear many of their experiences.” (Participant F)*

**TABLE 4:** Support: categories and subcategories.

Categories/subcategories	Representative codes
*Need for timely access to professional consultation/*	
Confirming with physicians about when to start weaning at follow‐up clinics	*“At the monthly pediatric follow-up, I checked to make sure it was okay to start weaning before I began.” (Participant C)*
Inability to consult timely at follow‐up clinics or health checkups	*“At the time of the 18-month checkup, his teeth had come in and he was already in the later weaning stage, so I wasn’t worried about feeding anymore.” (Participant B)*
Initial need to feel free to consult with local public health nurses, nutritionists, or visiting nurses	*“When I have something I’m a bit concerned about or want to talk over, I contact the public health nurse. She arranges a time that works for both of us, and we meet then to discuss my concerns.” (Participant E)*

*Exploration of approaches tailored to children’s condition with professionals/*	
Referral to nutritionists or physiotherapists by physicians or public health nurses depending on problem	*“Since I wasn’t sure how to proceed, I talked to the doctor, who then connected me to a nutritionist.” (Participant H)*
Sharing of feeding behavior with nutritionists, physiotherapists, or nursery teachers, followed by evaluation of chewing and swallowing	*“I showed the physical therapist a video of him chewing to check if he was eating properly. She said, ‘He’s chewing well, so maybe it’s time to try a thicker texture,’ and I’m gradually moving forward from there.” (Participant D)*
Receiving developmental tips from physicians, public health nurses, nutritionists, or physiotherapists based on children’s condition	*“The nutritionist told me that, overall, he wasn’t gaining weight because he wasn’t eating enough; he was about 60 kilocalories short, and his weight hadn’t increased at all.” (Participant H)*
Collaboration with daycare centers to tailor weaning feeding methods for children	*“At the daycare center, they were really thoughtful and tried different textures and consistencies every day. They shared with me what seemed to work best for my child at the time.” (Participant I)*

*Relief and confidence through collaboration with professionals/*	
Confidence provided by professionals’ assessment	*“When an expert says, ‘That’s fine,’ it reassures me that I’m not just being selfish—that I’m actually making decisions based on what’s best for my child. It gives me confidence and a sense of relief.” (Participant C)*
Relief provided by professionals’ continued attention to children’s development	*“Since the same people attend the baby food classes every month, I thought it was really nice to get ongoing advice from the same person, like, ‘Last time it was like this, so how’s it going recently?’” (Participant G)*
Trust in professionals who provide considerate consultation	*“The public health nurse assigned to my child listens to me, and I trust her enough to report everything to her after the follow-up visits.” (Participant E)*

### 3.1. Challenges: Difficulties in Deviating From Standard Weaning Practices

Although parents attempted to follow standard weaning practices, they encountered a mismatch between the expected developmental milestones based on corrected age and their infants’ actual developmental abilities. This category has six subcategories: (1) Difficulty deciding to start weaning based on criteria such as age or physical development, (2) Difficulty progressing to chewing or swallowing solid foods, (3) Anxiety during children’s vomiting or refusal during feeding, (4) Inability to progress according to corrected age, (5) Concern about effects of ingredients or amounts unsuitable for corrected age on children’s digestion or allergies, and (6) Difficulty judging whether information from peer support or internet is appropriate for children.

### 3.2. Challenges: Impatience With Children’s Growth and Development

Preterm infants are generally born significantly smaller and more physically immature than term infants; thus, parents expressed heightened concern regarding their children’s size and development, contributing to a significant psychological burden. This category has three subcategories: (1) Anxiety about whether children are gaining weight steadily along growth chart, (2) Depression arising from comparisons with full‐term children’s development, (3) Burden of feeding to support children’s growth.

### 3.3. Successes: Weaning Based on Children’s Actual Feeding Behavior

Parents considered their children’s motivation to feed crucial during the weaning process. During feeding difficulties, parents’ focus on their children’s actual feeding abilities was key to successful progress. This category comprises five subcategories: (1) Waiting for moment to start, trying not to miss children’s motivation to eat, (2) Relief that children do not dislike eating itself, (3) Identifying indicators other than age and valuing children’s pace, (4) Adjusting food texture and taste to what children can eat rather than age, and (5) Prioritizing children’s eating behavior over weight, with sufficient milk intake when weight gain is adequate.

### 3.4. Successes: Cooperation With Family and Experienced Caregivers

Although parents primarily managed the weaning process independently each day, sharing experiences with family members or peers enabled them to maintain a more positive outlook. This category involves three subcategories: (1) Uncertainty about how to start, leading to consultation with guidelines and experienced caregivers, (2) Proceeding in consultation with family, and (3) Sharing feeding difficulties in peer support settings, leading to positive reframing.

### 3.5. Support: Need for Timely Access to Professional Consultation

Given that feeding is a daily necessity, parents expressed a strong desire to consult with professionals promptly, either in advance or when difficulty arose. However, in preterm‐born children, the appropriate time for such consultation is often unclear. This category includes three subcategories: (1) Confirming with physicians about when to start weaning at follow‐up clinics, (2) Inability to consult timely at follow‐up clinics or health checkups, and (3) Initial need to feel free to consult with local public health nurses, nutritionists, or visiting nurses.

### 3.6. Support: Exploration of Approaches Tailored to Children’s Condition With Professionals

Given that feeding involves multiple interrelated factors, including oral motor function, physical development, nutritional needs, and environmental influences, parents actively sought to engage with professionals across various disciplines. This category has four subcategories: (1) Referral to nutritionists or physiotherapists by physicians or public health nurses depending on problem, (2) Sharing of feeding behavior with nutritionists, physiotherapists, or nursery teachers, followed by evaluation of chewing and swallowing, (3) Receiving developmental tips from physicians, public health nurses, nutritionists, or physiotherapists based on children’s condition, and (4) Collaboration with daycare centers to tailor weaning feeding methods for children.

### 3.7. Support: Relief and Confidence Through Collaboration With Professionals

Considering the uncertainty surrounding their preterm infants’ future development, parents experienced reduced anxiety through healthcare professionals’ ongoing support. This study comprises three subcategories: (1) Confidence provided by professionals’ assessment, (2) Relief provided by professionals’ continued attention to children’s development, and (3) Trust in professionals who provide considerate consultation.

## 4. Discussion

This study explored parents’ experiences during the weaning process of their preterm‐born children. As a result, these experiences were categorized into three stages: challenges, successes, and support. Understanding these experiences and incorporating them into NICU and follow‐up care may help develop staff education and establish support systems.

As a challenge during the weaning process, parents of preterm‐born children experienced “Difficulties in deviating from standard weaning practices.” Previous studies focusing on extremely preterm‐born children have similarly reported that parents experienced uncertainty and fear in the absence of guidelines [29]. Our findings further suggest that this difficulty arises because weaning does not progress according to age, partly due to developmental delays related to feeding. The immaturity of oral motor function [3] and early experiences of tube feeding in NICU [5] negatively affect the acquisition of feeding skills. Additionally, gross and fine motor development delays, which are frequently observed in preterm‐born children [30], may further hinder their ability to initiate and progress through the weaning process. These challenges are specific to preterm‐born children, highlighting the unique difficulties their parents face during the weaning process. Therefore, discharge planning from NICU should include anticipatory guidance on feeding development. By providing parents with information about potential delays and variations in weaning readiness they can form realistic expectations. From a nursing management perspective, standardized discharge planning requires nurse education that incorporates knowledge of the long‐term developmental trajectories and potential catch‐up patterns of preterm‐born children. In addition, structured monitoring and audit mechanisms—such as regular reviews of discharge planning documentation and tracking of post‐discharge developmental outcomes—are necessary to ensure consistent and appropriate implementation in clinical practice.

Moreover, parents identified “Weaning based on children’s actual feeding behavior” as a key to successful weaning. Previous studies have shown that parents’ responsiveness to children’s feeding cues is associated with increased food intake [31]. While close attention to the actual feeding behaviors of preterm‐born children is particularly important, most weaning guidelines use age as a primary reference [19]. For example, the Japanese guideline defines stages of feeding according to age (early stage at around 5‐6 months, middle stage at 7‐8 months, later stage at 9–11 months, and completion stage from 12 months onward) and suggests quantities and textures of solid foods for each stage [32]. However, feeding behavior is influenced by multiple interrelated factors, including motor development and sensory integration [11], which do not always align with age‐based stages in preterm‐born children [4]. Therefore, NICU follow‐up care should include individualized feedback to parents according to their child’s developmental readiness for weaning, rather than relying solely on age. Nursing management should ensure that follow‐up clinics allocate sufficient time for nurses to assess each child’s developmental readiness and parents’ concerns regarding feeding and parenting, thereby enabling nurse‐led consultations.

As a support, parents recognized “Need for timely access to professional consultation” and “Exploration of approaches tailored to children’s condition with professionals.” Previous studies have also emphasized the importance of professional support [29], and our findings further highlight the significance of timely and tailored consultation. Preterm birth is a major risk factor for pediatric feeding disorder (PFD) [33], which makes children 3.2 times more likely to refuse food and 2.7 times more likely to cough during feeding [34]. Early, tailored support is particularly crucial for children who struggle with feeding and for parents who witness these difficulties daily. These findings highlight the need to establish accessible consultation systems and implement the PFD screening tool [34] as part of early intervention strategies. To inform nursing management practices, community health centers should develop nurse‐led consultation pathways that provide timely access to support through home visits and child health checkups, while strengthening interprofessional networks for information sharing and coordinated referral.

Furthermore, parents felt “Impatience with children’s growth and development” during the weaning process. Feeding is a critical factor directly linked to preterm‐born children’s growth. Previous studies have described feeding as an emotional experience for parents of preterm‐born children [35], and have reported that adherence to age‐appropriate growth charts can provide reassurance [29]. In contrast, in this study, parents described feelings of impatience even while closely monitoring growth charts. To cope with these feelings, parents appeared to proceed positively by “Cooperating with family and experienced caregivers” and felt “Relief and confidence through collaboration with professionals.”

While these findings are partially consistent with previous studies on general weaning [36], this study also highlights characteristics unique to parents of preterm‐born children, including the need for peer support and ongoing professional involvement. Family‐centered care emphasizes collaborative relationships and information sharing between professionals and families [20], thereby key to enhancing parenting confidence [37]. In Japan, the Maternal and Child Health Handbook, which serves as a tool for sharing health and developmental information between parents and professionals, has recently been updated to include growth charts adapted for preterm infants [38]. After NICU discharge, continued community‐based support—facilitated by such tools for communication and developmental monitoring—can help maintain collaboration between healthcare professionals and parents and foster parental confidence over time. From a care coordination perspective, establishing information sharing systems among NICU, follow‐up clinics, and community health settings is necessary to ensure continuity of support after discharge.

### 4.1. Limitations

This study has some limitations. First, the findings were based on interviews with nine mothers in Japan who were recruited through multiple family support groups. The perspectives of fathers, grandparents, and parents who were not connected to support groups were not included. In this study, purposive sampling was employed to enhance the credibility and transferability of the findings. Although the sample size was determined based on a previous study [28], different codes might have emerged depending on participants’ backgrounds, such as medical accessibility and socioeconomic status, which were not collected in this study. Consequently, the extent to which lower‐resource families were represented could not be determined. Additionally, parenting stress varies across countries [39]. Participants’ weaning experiences may have been influenced by sociocultural factors in Japan, such as the declining birth rate and the increasing prevalence of nuclear families.

Second, none of the participants’ children required tube feeding or had unstable respiratory conditions. As these clinical factors significantly affect the feeding process [5, 6], the weaning experiences reported in this study may not be transferable to parents in other clinical contexts.

## 5. Conclusion

Parents of preterm‐born children experienced unique challenges during the weaning process following NICU discharge. Timely professional consultation and individualized assistance in addressing feeding‐related challenges according to each child’s developmental status are necessary. Nurses in hospitals and community health centers play a critical role in supporting parents during the weaning process. By understanding parents’ perspectives and experiences, they can offer tailored consultation on weaning and facilitate collaboration with interdisciplinary teams. Additionally, continued community‐based support throughout the weaning process may help enhance parental confidence over time.

### 5.1. Implications for Nursing Management

The findings of this study on weaning among preterm‐born children have important implications for nursing management from a family‐centered care perspective. It is essential for nursing management to establish a coordinated support system that spans the NICU and community healthcare settings to facilitate a smooth transition from breastfeeding to weaning. This includes discharge planning, follow‐up assessments of feeding development, and nurse education on both the long‐term developmental needs of preterm‐born children and family empowerment strategies. Furthermore, nursing management should support the establishment of accessible parent consultation pathways within community healthcare settings, enabling parents to seek routine advice. The support system should also facilitate timely information sharing and interdisciplinary collaboration tailored to the needs of each child and family.

## Author Contributions

Maki Fujitsuka: Conceptualization, Methodology, Investigation, Data curation, Formal analysis, Writing – original draft. Shingo Ueki: Conceptualization, Methodology, Formal analysis, Writing – review & editing, Supervision. Seiichi Morokuma: Conceptualization, Writing – review & editing, Supervision.

## Funding

This study did not receive any funding.

## Conflicts of Interest

The authors declare no conflicts of interest.

## Data Availability

The data that support the findings of this study are available from the corresponding author upon reasonable request.
